# Coroner for Middlesex

**Published:** 1830-10-01

**Authors:** 


### CORONER FOR MIDDLESEX.

THIS energetic contest has ended, after a tremendous expenditure of money —a most exuberant exhibition of zeal on the part of the friends—a display of no mean talent on the part of one of the candidates—and a degree of excitement among the people seldom exceeded by the most contested parliamentary election. We have neither time nor space for a disquisition on the qualifications necessary for the office of coroner. Our own impression is, that a certain amount, both of medical and legal knowledge, is necessary—but that the quantum of either need not be very great. We imagine that it would be much easier for the medical man to acquire the requisite modicum of law, than for the lawyer to furnish himself with the proper proportion of physic—and hence, upon the whole, we are inclined to advocate the cause of medical coroners—seeing the small allotment of loaves and fishes which the medical practitioner can expect, beyond the direct drudgery of his own profession. If we could trust to the public—or at least the popular feeling respecting this litigated point, as expressed during the recent election, we might confidently expect that medical coroners would, in future, be elected—at least in Middlesex. But we cannot flatter our brethren that the popular feeling breathed forth so ardently at Clerkenwell, was entirely divested of politics. Independent of the *medical* party which was actively and zealously employed in furtherance of the medical candidate's interest, the speeches on the hustings by Mr. Wakley himself, made a powerful impression on the popular mind. Arts, manœuvres, and language seem to be allowed at elections, which would not be borne in private life, or civil intercourse; and on these we are not inclined to remark. Certes we cannot approve of all that have been put in force during the late contest. But, from what we have seen of Mr. Wakley's talents for working on the popular mind, we strongly advise him to contest the next Westminster election with Sir Francis Burdett. The impression which has been made on the public, by the recent exposé of the worthy Baronet's common sense, in St. John Long's case, will be an admirable and potent adjuvant to Mr. Wakley, in a popular contest for a seat in parliament. We very much doubt whether Mr. Wakley would not beat out of the field, his friend Mr. Hume, in the event of a vacancy for Middlesex. Should a revolution ever take place in this country, (which Heaven avert) we shall be much surprised if Mr. Wakley does not figure conspicuously on the political arena. Few men are better calculated to call forth popular feeling—but whether the effervescence of popular feeling would be governed by reason, and directed to beneficial purposes, we have great doubts.

But politics apart, the unsuccessful candidate for coroner, in the vacancy of Mr. Unwin, has a very fair chance of success, in the event of Mr. Stirling's resignation—a resignation which senectitude, with its concomitant infirmities, ought to point out. It is fortunate, in one sense, and unfortunate in another, that AGE, which plucks from us the feathers of early acquirement—saps the energies of our faculties—renders us a wreck on the stream of TIME,—and ultimately plunges us in the gulf of everlasting oblivion, rarely deprives us of the fond idea that we are still possessed of intellectual vigour, however large the "youthful hose" may appear for our "shrunk shanks"—or the "spectacles on nose, and pouch on side," may indicate the propriety of retiring with, "the lean and slippered pantaloon," from the gaze and pity—too often the contempt, of those who are rapidly following in the same track!

